# Evidence summary on perioperative blood glucose management in liver cancer patients

**DOI:** 10.3389/fonc.2026.1790506

**Published:** 2026-07-21

**Authors:** Yuxiao Li, Ming Pang, Caifang Gong, Chuan You

**Affiliations:** Department of Hepatobiliary Surgery I, Affiliated Hospital of North Sichuan Medical College, Nanchong, Sichuan, China

**Keywords:** evidence synthesis, evidence-based, glycemic control, hepatocellular carcinoma, perioperative period

## Abstract

**Objective:**

Systematically retrieve, evaluate, and synthesize the best available evidence on perioperative glycemic management in liver cancer patients to provide evidence-based guidance for clinical nursing practice.

**Methods:**

Using the “6S” evidence model, we conducted a top-down search of domestic and international databases, guideline portals, and professional association websites for best clinical practices, guidelines, systematic reviews, evidence summaries, expert consensus statements, and randomized controlled trials concerning perioperative glycemic management in hepatocellular carcinoma patients. The search period spanned January 2015 to December 2025. Selected literature underwent quality assessment, evidence extraction, and synthesis.

**Results:**

A total of 14 documents were included, comprising 4 guidelines, 1 systematic review, 1 evidence summary, 3 expert consensus statements, and 5 randomized controlled trials. This yielded 28 best evidence items across six domains: team-based perioperative glycemic management for liver cancer patients, preoperative assessment management, medication and nutritional management, blood glucose monitoring and targets, complication management, and health education.

**Conclusion:**

This study summarizes the best available evidence for perioperative glycemic management in patients with hepatocellular carcinoma, providing evidence-based guidance for healthcare professionals. Each piece of evidence should be thoroughly assessed for its appropriateness and feasibility before being translated and applied in clinical practice.

## Introduction

Primary liver cancer (PLC) ranks as the sixth most common cancer worldwide, and liver resection remains one of the primary curative treatments for this disease (1).However, liver resection procedures are complex, highly invasive, and lengthy. Patients often present with varying degrees of hepatic insufficiency, making them prone to perioperative stress-induced hyperglycemia. Postoperative complications can reach rates as high as 18.9% to 53.3% (2).The liver is closely associated with glucose metabolism. Hepatic glycogen serves as the primary direct source of blood glucose during fasting states, and the liver also functions as a crucial target organ for insulin action, playing a key role in maintaining blood glucose homeostasis. When liver function is impaired, its ability to regulate blood glucose significantly declines, leading to impaired glucose metabolism. This is closely associated with perioperative complications. Prolonged hyperglycemia directly impairs liver regeneration, resulting in delayed wound healing, hyperosmolar syndrome infections, liver failure, and even death (3, 4). Hypoglycemia is one of the clinical manifestations of the paraneoplastic syndrome associated with hepatocellular carcinoma. It is often overlooked due to being masked by symptoms of liver cancer and can be mistaken for hepatic encephalopathy, leading to missed opportunities for optimal treatment (5). Additionally, elevated postoperative fasting blood glucose levels also constitute an independent risk factor for recurrence following liver cancer surgery (6–8). Therefore, perioperative glycemic management is particularly critical for patients with hepatocellular carcinoma. Currently, evidence regarding perioperative glycemic management in these patients remains fragmented both domestically and internationally, lacking unified standards and guidelines that hinder effective clinical practice. This study synthesizes relevant evidence on perioperative glycemic management for hepatocellular carcinoma patients worldwide, aiming to provide evidence-based support for standardizing clinical glycemic management.

This study has been registered with the Evidence-Based Nursing Center at Fudan University (Registration Number: ES20259043).

## Materials and methods

### Retrieval strategy

Following the top-down principle of the “6S” evidence resource pyramid model, a combination of subject headings and free-text terms was employed to sequentially search the following databases: BMJ Best Practice, UpToDate, the JBI Centre for Evidence-Based Healthcare, the World Health Organization (WHO), the Australian Diabetes Association website, the British Diabetes Association website, the Guidelines International Network (GIN), Scottish Intercollegiate Guidelines Network (SIGN), New Zealand Guidelines Group (NZGG), China Medical Guide Network, PubMed, Embase, SinoMed, China National Knowledge Infrastructure (CNKI), and Wanfang Database. English search terms: “Carcinomas, Hepatocellular/Hepatocellular Carcinomas/Hepatoma/Adult Liver Cancer, ” “Perioperative Periods/Operative Procedures/Surgical Procedures/Hepatectomy, ” “Blood Glucose/blood glucose management/glycemic control/Hypoglycemia/Hyperglycemias.” The search timeframe spanned from January 2015 to December 2025.

**Figure 1:**
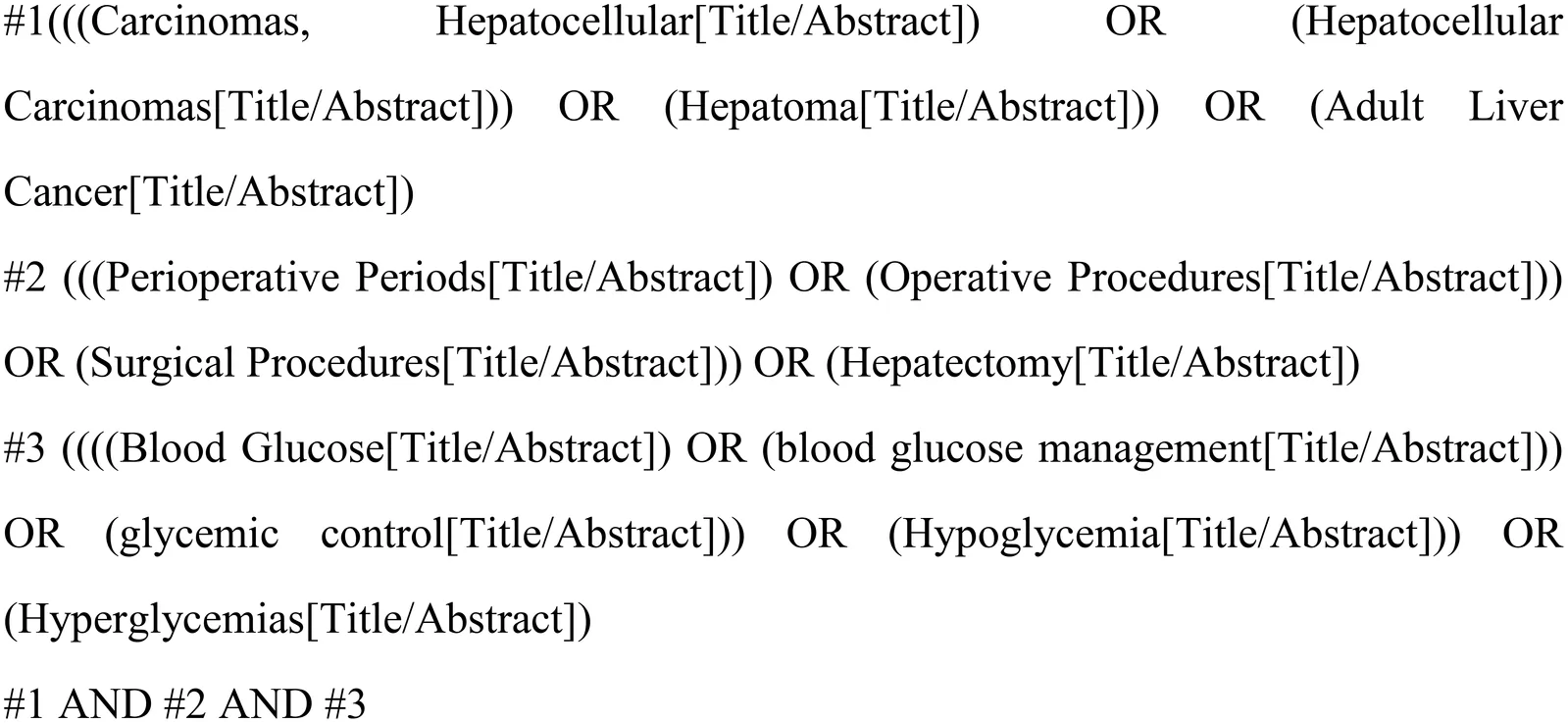
PubMed search strategy for evidence retrieval.

Using PubMed as an example, the search query is shown in **Figure 1**.

### Literature inclusion and exclusion criteria

Constructing evidence-based questions based on the PIPOST model (9).Evidence Application Target Population (P): Evidence specifically addressing liver resection patients was preferentially included. When liver-specific evidence was unavailable, high-quality evidence from adult perioperative glycemic management guidelines, consensus statements, and related hepatobiliary populations was considered for evidence supplementation and contextual adaptation; Intervention (I): Comprehensive measures for perioperative blood glucose management in hepatocellular carcinoma patients; Professionals Applying Evidence (P): Clinical healthcare providers; Outcome (O): Blood glucose control levels, incidence of glucose-related complications, incidence of abnormal blood glucose levels, etc.; Setting (S): Home, hospital; Type of Evidence (T): Clinical decision aids, guidelines, expert consensus statements, evidence summaries, systematic reviews, randomized controlled trials, etc.

Inclusion criteria for this study’s literature: ① Patients undergoing liver resection as research subjects; ② Research content covering screening, assessment, diet, exercise, pharmacotherapy, and monitoring targets for glycemic management; ③ Evidence types including clinical decision aids, guidelines, evidence summaries, expert consensus statements, systematic reviews, and randomized controlled trials.

Exclusion criteria: ① Non-Chinese or non-English literature; ② Low methodological quality; ③ Duplicate publications; ④ Inaccessible full texts or missing information.

### Literature screening and data extraction

Two researchers trained in evidence-based nursing methodology independently assessed the included literature. When disagreements arose during evaluation, the research team members collectively discussed inclusion criteria and ultimately reached a consensus decision.

### Literature quality evaluation

Different evaluation tools are used based on the type of literature. Clinical decision-making: Quality assessment tools for evidence summaries are employed (10). Guidelines: Quality assessment using the AGREE II guideline evaluation tool (11), which consists of 23 items across six domains. Each item was scored on a 7-point Likert scale (1 = strongly disagree to 7 = strongly agree). The standardized score for each domain was calculated as follows: Domain score = (Obtained score − Minimum possible score)/(Maximum possible score − Minimum possible score) × 100%.The obtained score represented the sum of all reviewers’ ratings for the items within a given domain. Based on the standardized scores of the six domains, guidelines were classified into three recommendation grades: Grade A, all six domains scoring >60%; Grade B, three or more domains scoring between 30% and 60%; and Grade C, three or more domains scoring <30%. Guidelines graded as C were excluded from evidence synthesis. Evidence synthesis: Evaluation using the CASE methodology and reporting assessment (10), requiring retrospective reference tracking and application of corresponding study quality assessment tools. Systematic reviews: Utilizing the JBI methodology quality assessment tool (12). Expert consensus statements: Evaluate using the JBI Methodological Quality Assessment Tool for Expert Opinion and Professional Consensus Articles (13). Randomized Controlled Trials: Evaluate using the Cochrane Collaboration Risk of Bias Assessment Tool (14).

### Evidence extraction, integration and evaluation

Two researchers independently extracted and synthesized the included evidence, with a third researcher conducting verification. The evidence synthesis principles followed in this study were: ① When evidence on the same topic was identical or complementary, evidence conforming to professional terminology and linguistic logic was selected for integration; ② When evidence content was inconsistent, the principles of prioritizing high-quality evidence, prioritizing the most recently published authoritative literature, and prioritizing evidence-based evidence were adhered to. Evidence was graded into five levels based on study design type, using the 2014 Australian JBI Evidence Grading System (15). Level 1 represents the highest quality, while Level 5 denotes the lowest quality. Recommendation grades were determined according to the JBI FAME framework, which considers Feasibility, Appropriateness, Meaningfulness, and Effectiveness. Therefore, recommendation grades were assigned based not only on the level of evidence but also on the overall balance of benefits, clinical applicability, feasibility of implementation, and patient relevance. Recommendations were categorized as Grade A (strong recommendation) or Grade B (weak recommendation).

### Assessment of evidence transferability and applicability

Given the limited availability of high-quality evidence specifically addressing perioperative glycemic management in patients undergoing liver resection, a transferability-based evidence synthesis strategy was adopted. Liver surgery–specific evidence was prioritized whenever available. For evidence domains where liver-specific recommendations were lacking, high-quality guidelines and consensus statements developed for adult perioperative patients with diabetes were incorporated. The transferability of these recommendations was assessed based on similarities in target population characteristics, intervention mechanisms, and clinical settings. Recommendations regarding blood glucose monitoring, insulin adjustment, hypoglycemia prevention, ketone monitoring, and perioperative glycemic targets were considered transferable because their physiological rationale is not specific to liver surgery but reflects general principles of perioperative glucose management. Evidence from these indirect sources was then reviewed and contextualized alongside available liver surgery-specific literature before being incorporated into the final evidence synthesis.

## Results

This study initially screened 2, 916 relevant publications. After removing duplicate publications, 2, 400 articles remained. Following exclusion of non-compliant studies based on title and abstract review, 68 articles were retained. Full-text review further reduced the pool to 36 articles. Low-quality studies were excluded based on quality assessment results, ultimately yielding 14 included publications. The final dataset comprised 4 guidelines (16, 19), 1 systematic review (20), 1 evidence summary (21), 3 expert consensus statements (22, 24), and 5 randomized controlled trials (25, 29). Basic characteristics of the included studies are summarized in **Table 1**.

**Table 1:** Basic characteristics of the included literature (n = 14).

Included literature	Year	Source	Type of literature	Topic of literature
Diabetes Australia (16)	2022	Diabetes Australia	Guideline	Management of Perioperative Diabetes or Hyperglycemia in Adults
CPOC Diabetes Guidelines Working Group (17)	2023	Centre for Perioperative Care	Guideline	Perioperative Care for Diabetic Patients Undergoing Elective and Emergency Surgery
Chinese Branch of the International Association for the Study of the Liver, Pancreas, and Gallbladder, etc. (18)	2025	Wanfang Data	Guideline	Multidisciplinary Clinical Management Guidelines for the Perioperative Period of Hepatectomy (2025 Edition)
Joliat GR et al. (19)	2022	PubMed	Guideline	Perioperative Care for Liver Surgery
Wu Ping et al. (20)	2025	PubMed	Systematic review	Effects of Different Preoperative Carbohydrate Loading Protocols on Insulin Resistance
Zhou Yifeng et al. (21)	2024	CNKI	Evidence summary	Evidence Synthesis on Goal-Oriented Glucose Monitoring in Patients Undergoing Hepatocellular Carcinoma Resection Surgery
Chinese Society of Parenteral and Enteral Nutrition, Chinese Medical Association, etc. (22)	2025	Wanfang Data	Expert consensus	Nutritional Support Management for Patients with Hyperglycemia in the Perioperative Period
Chen Liming et al. (23)	2021	Wanfang Data	Expert consensus	Expert Consensus on Perioperative Blood Glucose Monitoring in Adults
Nursing Subcommittee of the Interventional Medicine Branch, Hubei Medical Association (24)	2024	Wanfang Data	Expert consensus	Perioperative Glycemic Management in Hepatocellular Carcinoma with Diabetes Treated by Transarterial Chemotherapy Embolization
Kumar M et al. (25)	2025	PubMed	RCT	Preoperative carbohydrate loading can reduce perioperative insulin resistance in the liver.
Lin Chunhui et al. (26)	2024	Wanfang Data	RCT	Multidisciplinary Collaboration Combined with Informatized Blood Glucose Management in the Perioperative Period for Hepatocellular Carcinoma Patients: An Evaluation of Clinical Outcomes
Zhu Yi et al. (27)	2015	万方	RCT	Effects of Different Doses of Ustilaginine on Preventing Insulin Resistance in Patients Undergoing Hepatic Partial Resection
Li Jianling et al. (28)	2020	CNKI	RCT	The Effect of Dexmedetomidine on Intraoperative Stress Responses in Elderly Patients Undergoing Hepatocellular Carcinoma Resection
Chen Cui et al. (29)	2016	CNKI	RCT	Dietary Guidance Based on Glycemic Index for Patients with Primary Liver Cancer and Diabetes

### Quality evaluation results of the included literature

#### Quality evaluation results of the guidelines

A total of 4 guidelines were included. The quality assessment results are shown in the table. All guidelines received a “Yes” rating for the question “Would you recommend this guideline?” Overall quality was high, and they were included in **Table 2**.

**Table 2:** Quality evaluation results of the guidelines (n = 4).

Included literature	Standardized scores in various domains (%)						≥%)r	≤%)r	Quality evaluation
	Scopes and objects	Stakeholder Particip-ant	Rigor of development	Clarity of guidelines	Applicability	Editorial independence			
Diabetes Australia (16)	72.2%	72.2%	61.9%	94.4%	85.7%	57.1%	5	0	B
CPOC Diabetes Guidelines Working Group (17)	83.3%	94.4%	69.0%	88.9%	87.5%	41.7%	5	0	B
Chinese Branch of the International Association for the Study of the Liver, Pancreas, and Gallbladder, etc. (18)	95.2%	85.7%	58.9%	95.2%	21.4%	85.7%	4	1	B
Joliat GR et al. (19)	94.4%	61.1%	77.1%	94.4%	58.3%	75.0%	5	0	B

#### Quality evaluation results of systematic reviews

Included in 1 systematic review (20), with all quality assessment items rated as “yes.” The literature is of high quality and is therefore included.

#### Quality assessment results of evidence synthesis

Included in 1 evidence summary (21). Except for the evaluation item “Was the review/editing of the evidence summary transparent?” which was rated as “Partially, ” all other items were rated as “Yes.” The literature quality was high, and it was therefore included.

#### Quality evaluation results of expert consensus statements

Included in 3 expert consensus documents (22, 24), all evaluation criteria were met (“Yes”), and the overall quality of the consensus was high; all were included.

#### Quality assessment results of randomized controlled trials

Five randomized controlled trials were included (25–29). Among these, one trial (25) had “high risk of bias” for “blinding of participants and personnel” and “unclear risk of bias” for “blinding of outcome assessors”. one trial (27) had “unclear risk of bias” for “concealment of allocation” and “blinding of participants and personnel”, and three trials (26, 28, 29) had “unclear” and “high risk of bias” for “allocation concealment” and “blinding of participants and personnel”; all other domains were rated as “low risk of bias.”

### Summary and description of evidence

Through evidence extraction and synthesis, 28 evidence points were compiled across six key areas: team building management, preoperative assessment management, medication and nutrition management, blood glucose monitoring and targets, complication management, and health education, as shown in **Table 3**.

**Table 3:** Evidence summary on perioperative blood glucose management in liver cancer patients.

Evidence item	Evidence content	Evidence level	Recommendation level
team-based perioperative glycemic management	1. Supported by a team of diabetes specialists, it helps standardize blood glucose monitoring and intervention (21).	5	B
	2. Establish a multidisciplinary blood glucose management team led by hepatobiliary surgery, incorporating endocrinology, anesthesiology, nutrition, rehabilitation, and nursing teams, and provide specialized training for medical staff across relevant departments (21, 23, 26).	1	B
	3. Utilizing an information-based blood glucose management system enables real-time monitoring, sharing, and automated alerts for perioperative blood glucose data (26).	1	B
preoperative assessment management	4. Upon admission, liver cancer patients should undergo point-of-care testing (POCT), fasting blood glucose, and HbA1c assessments to evaluate baseline glycemic control and antidiabetic medication use. A comprehensive evaluation should include liver disease status, Child-Pugh classification, severity of hepatitis or cirrhosis, AFP levels, and end-organ damage (21).	3	B
	5. Preoperative HbA1c testing is recommended to assess glycemic control over the past 3 months, supplemented by venous fasting blood glucose (FBG) and capillary blood glucose (CBG) to evaluate perioperative glucose levels. HbA1c ≥bA mmol/mol (6.5%) indicates diabetes, while HbA1c ≥bA mmol/mol (9.0%) suggests poor glycemic control. Elective surgery should be postponed to allow for glycemic optimization. Preoperative HbA1c should be optimized to <69 mmol/mol (8.5%) whenever possible. Values >69 mmol/mol (8.5%) warrant specialist consultation (16, 17) (21, 23, 24)	5	B
	6. All diabetic patients should have a clear, written perioperative blood glucose management plan established prior to surgery, including fasting arrangements, adjustments to hypoglycemic medications, blood glucose monitoring frequency, and protocols for managing abnormal blood glucose levels (16).	5	A
	7.Patients with perioperative hyperglycemia should receive the same level of perioperative blood glucose monitoring and management as those with known diabetes (16).	5	A
medication and nutritional management	8. Adjust hypoglycemic therapy during the perioperative period based on drug type. All non-insulin hypoglycemic agents should be discontinued on the day of surgery. Among these, SGLT2 inhibitors should be stopped at least 2 days prior to surgery and on the day of surgery to reduce the risk of normal glycemic ketoacidosis. Continue basal insulin therapy perioperatively; avoid complete discontinuation of insulin. If patients are at risk for nocturnal or recent hypoglycemia, reduce basal insulin dosage by approximately 20% (16).	5	B
	9. Variable Rate Intravenous Insulin Infusion (VRIII) is prioritized for: skipping >1 meal, T1DM without basal insulin, HbA1c >6.9% (8.5%), most emergency cases, and persistent perioperative hyperglycemia. The recommended fluid for the VRIII bypass is 5% glucose + 0.45% saline + KCl; alternating 5% glucose and 0.9% saline based on blood glucose levels is not recommended (17).	5	B
	10. Intravenous administration of 2500 U/kg of ulinastatin prior to anesthesia induction and at the start of surgery in patients undergoing partial hepatectomy effectively prevents intraoperative insulin resistance (27).	1	B
	11.For elderly patients undergoing liver resection, the intraoperative use of dexmedetomidine may help stabilize the stress response and thereby provide some assistance in controlling post-operative blood glucose levels (28).	1	B
	12.The stress hyperglycemia ratio (SHR) can serve as an indicator for monitoring blood glucose levels in perioperative patients during nutritional support therapy (22).	5	B
	13.For patients with perioperative hyperglycemia undergoing nutritional support, continuous insulin infusion via an intravenous infusion pump is an ideal strategy (19, 22).	5	B
	14. For patients undergoing planned liver resection, minimize fasting duration by implementing a 6-hour preoperative fasting period and a 2-hour fluid restriction. Providing moderate carbohydrate beverages the night before surgery and 2 to 4 hours prior to anesthesia induction improves postoperative glucose control and significantly reduces perioperative insulin resistance compared to traditional overnight fasting (16–20, 22, 25)	1	A
blood glucose monitoring and targets	15.Appropriate blood glucose monitoring methods should be selected for different perioperative phases, with point-of-care testing (POCT) being the preferred choice, particularly for hemodynamically stable patients. Monitoring frequency should be intensified during surgery and in the early postoperative period, and may be appropriately reduced once blood glucose levels stabilize (16, 21, 23).	5	A
	16.During the perioperative period, ensure safe handover procedures between the operating room, anesthesia recovery room, and patient ward. Maintain complete documentation of blood glucose monitoring results, medication administration, and relevant medical orders (21).	5	B
	17. Develop personalized blood glucose monitoring protocols. Blood glucose monitoring is performed upon patient admission, on the morning of surgery, during surgery, and upon return to the ward postoperatively. For patients with abnormal blood glucose levels, monitoring is conducted every 30–60 minutes during surgery and every 1–2 hours postoperatively. In cases of abnormal blood glucose fluctuations, monitoring frequency should be increased as necessary based on blood glucose levels, or CGM should be employed. This should be combined with blood gas analysis to ensure effective management of blood glucose, electrolytes, and acid-base balance (18, 21, 22, 26)	1	A
	18. Intraoperative blood glucose targets should be maintained between 6.0–10.0 mmol/L, with routine point-of-care testing (POCT) monitoring every 2 hours (17, 21, 24).	1	B
	19. Minimize blood glucose fluctuations in patients with perioperative stress hyperglycemia (SH) during nutritional support therapy, maintaining blood glucose levels between 8.0–10.0 mmol/L (22).	5	B
	20. The target blood glucose level within 3 days postoperatively should be <12.0 mmol/L (21).	5	B
complication management	21.Definition of hypoglycemia: CBG <4 mmol/L; For individuals with lower hypoglycemia risk managed by diet and medication: Recommended target range is 4–12 mmol/L; For most patients using hypoglycemic agents/insulin: Intervention should be considered at CBG <6.0 mmol/L to prevent hypoglycemia (16, 17).	5	B
	22. Hypoglycemia is a significant perioperative risk. When hypoglycemia is suspected during the perioperative period, point-of-care testing (POCT) should be performed immediately and reported to the physician. For patients with confirmed hypoglycemia, monitoring intervals should be shortened based on clinical condition until blood glucose is corrected (21, 24).	1	A
	23. Intervention should be considered when blood glucose exceeds 10 mmol/L. Discontinue VRIII and treat hypoglycemia when glucose falls below 6 mmol/L, then restart within 20 minutes. If glucose remains above 12 mmol/L for three consecutive readings with insufficient decline, increase the infusion rate. Reduce the VRIII rate if hypoglycemia risk factors are present (16, 17).	5	B
	24. When hypoglycemia is confirmed by a conscious individual, immediately administer 15 g of carbohydrates. Perform a point-of-care test (POCT) after 15 minutes. If blood glucose remains uncorrected, repeat the above measures. If blood glucose has been corrected to 3.9 mmol/L or above but the next meal is more than 1 hour away, continue providing starchy or protein-containing foods (21).	5	B
	25.If the patient experiences discomfort or persistent hyperglycemia (>13 mmol/L on two or more consecutive occasions), measure capillary blood ketones. For patients regularly using SGLT2 inhibitors, daily blood ketone monitoring is recommended during hospitalization (17).	5	B
	26.When blood glucose exceeds 13.9 mmol/L or symptoms such as nausea and vomiting occur, immediately notify the physician for intervention and perform POCT testing once every 30 minutes (21).	5	B
health education	27.For patients with primary liver cancer and diabetes undergoing surgery, implementing glycemic index (GI)-based dietary guidance in addition to conventional dietary advice improves glucose metabolism control during the 1–3 months post-surgery (29).	1	B
	28. Diabetes education for patients and their families is provided throughout the perioperative period (23).	5	A

## Discussion

Multidisciplinary teams are essential for effective blood glucose management

Perioperative glycemic management for liver cancer requires coordinated communication across multiple disciplines (23). Establishing a multidisciplinary glycemic management team led by hepatobiliary surgeons and including endocrinology, anesthesiology, nutrition, rehabilitation, and nursing staff, coupled with specialized training for relevant departmental healthcare providers, is crucial for improving perioperative glycemic control in liver cancer patients (21, 26). Currently, inpatient blood glucose management in China predominantly relies on traditional models—departmental self-management and endocrinology consultations—which often result in inadequate specialized health education and low blood glucose control rates (30). Consequently, multiple studies (26, 30, 31) advocate for adopting an information-based blood glucose management model, which holds greater promise and applicability for achieving real-time monitoring, data sharing, and automated alerts for perioperative blood glucose data.

### Preoperative assessment management is the prerequisite for developing treatment plans

Preoperative systematic assessment of glycemic status and associated risks aids in identifying high-risk individuals and guiding glycemic management strategies. Compared to a single blood glucose reading, evaluating glycated hemoglobin (HbA1c) better reflects a patient’s average blood glucose levels over the preceding 2–3 months (24). Liver cirrhosis, Child-Pugh classification, and chronic inflammation can disrupt glucose metabolism, further increasing perioperative glycemic risks in hepatocellular carcinoma patients (32). Therefore, it is recommended to conduct systematic, comprehensive assessments of hepatocellular carcinoma patients upon admission and preoperatively. Based on integrated evaluation of blood glucose, liver function, and comorbid conditions, risk stratification should be performed to develop personalized perioperative glycemic management plans, ensuring stable glycemic transition throughout the perioperative period.

### Medication and nutritional support for stable blood glucose control

Perioperative blood glucose fluctuations in hepatocellular carcinoma patients are not only related to baseline glucose metabolism but also influenced by perioperative medications, nutritional intake, and surgical stress. However, there are currently no universal guidelines or standards for pharmacological glucose control during the perioperative period in these patients. Commonly used clinical treatments include oral hypoglycemic agents or insulin therapy. Evidence (16, 17) emphasizes safe transition as a core principle, recommending standardized preoperative discontinuation of non-insulin medications and personalized intraoperative insulin dose adjustments. It also clarifies the indications and treatment protocols for VRIII use, underscoring the importance of continuous perioperative blood glucose management. Existing evidence (16, 19, 20, 22, 25) recommends modified preoperative gastrointestinal preparation protocols that effectively alleviate preoperative hunger and thirst while reducing perioperative insulin resistance incidence, challenging traditional approaches.

### Personalized dynamic monitoring and phased goal management

Dynamic monitoring and phased target control are integral throughout the entire perioperative period in glycemic management. An accurate, rapid, and feasible blood glucose monitoring plan is a prerequisite for effective glycemic control (33). Evidence (16, 21, 23) establishes POCT as the foundational monitoring method, though monitoring frequency requires dynamic adjustment based on the patient’s physiological state and surgical progress. The seamless handover of postoperative monitoring data and medical orders across departments for hepatocellular carcinoma surgery patients is crucial for precise glycemic control and further ensures continuity of care (21). Evidence (17, 21, 24) generally advocates setting differentiated blood glucose monitoring targets based on the pathophysiological characteristics of different perioperative phases. For example, intraoperative blood glucose control targets can be maintained at 6–10 mmol/L without overly stringent requirements to avoid hypoglycemia caused by excessive tightness. Early postoperative targets can be moderately relaxed to <12 mmol/L to safely navigate the acute stress period.

### Prevention-oriented complication management

Patients with hepatocellular carcinoma frequently experience hypoglycemic episodes due to factors such as reduced hepatic glycogen reserves and diminished insulin inactivation capacity (34). Therefore, healthcare providers should closely monitor for hypoglycemia in addition to assessing abnormal hyperglycemia. Included studies (16, 17, 21, 24) have established protocols for managing critical hyperglycemic and hypoglycemic emergencies, providing evidence-based guidance for preventing abnormal blood glucose levels and related complications during the perioperative period of liver cancer. Relevant evidence (23, 29) demonstrates that personalized dietary guidance combined with health education for liver cancer patients with diabetes enables a multimodal prevention strategy, promoting rapid postoperative recovery.

Several limitations should be acknowledged. First, the included evidence was heterogeneous with respect to study design, target populations, and clinical settings. In addition to liver resection–specific studies, some recommendations were derived from clinical practice guidelines, expert consensus statements, and evidence from broader perioperative diabetes populations when direct evidence was unavailable. Although transferability was carefully assessed, the applicability of certain recommendations to patients with liver cancer undergoing hepatectomy may remain limited. Second, several recommendations were supported primarily by expert consensus or indirect evidence, and therefore should be interpreted with appropriate caution. Finally, some randomized controlled trials included in the evidence synthesis had methodological limitations or potential risks of bias, which may affect the certainty of the evidence. Future high-quality studies focusing specifically on perioperative glycemic management in patients undergoing liver resection are warranted.

## Conclusion

This study synthesizes the best available evidence for perioperative glycemic management in hepatocellular carcinoma patients, covering six key areas: team-based management, preoperative assessment management, medication and nutritional management, glucose monitoring and targets, complication management, and health education. It provides evidence-based guidance for healthcare professionals developing perioperative glycemic management strategies for hepatocellular carcinoma. However, some evidence originates from international sources. When applying this evidence, healthcare providers should assess its adaptability and feasibility within the context of China’s patient population characteristics and clinical settings.
